# FAP Serves as a Prognostic Biomarker in Head and Neck Squamous Cell Carcinoma

**DOI:** 10.1155/2024/8810804

**Published:** 2024-05-24

**Authors:** Zhanpeng Liao, Haidong Fan, Junquan Weng, Jieyu Zhou, Yuyan Zheng

**Affiliations:** Department of Stomatology, Shenzhen People's Hospital (The Second Clinical Medical College, Jinan University; The First Affiliated Hospital, Southern University of Science and Technology), Shenzhen 518020, Guangdong, China

## Abstract

Head and neck squamous cell carcinoma (HNSCC) poses significant challenges with poor survival rates and limited therapeutic strategies. Our study, using The Cancer Genome Atlas (TCGA) data, assesses cancer-associated fibroblast (CAF) gene signatures' clinical relevance. In our analysis across TCGA tumor types, differential gene expression analysis revealed that fibroblast activation protein (FAP) is upregulated in tumor tissues and associated with poorer survival rates in HNSCC. Furthermore, mechanistic studies employing gene-silencing techniques substantiated that FAP knockout led to a significant decrease in cellular proliferation, invasion, and migration in HNSCC cell lines. Through Gene Ontology and Kyoto Encyclopedia of Genes and Genomes enrichment analyses, we established that high FAP expression correlates with vital biological processes such as extracellular matrix organization, angiogenesis, and cellular motility. Importantly, FAP was found to regulate these processes by promoting the expression of key proteins involved in epithelial–mesenchymal transition-related pathways. Additionally, our analysis revealed a significant correlation between FAP expression and the expression profiles of immune checkpoint molecules, underscoring its potential role in immune modulation. Collectively, our findings illuminate FAP's pivotal role in HNSCC pathogenesis and its potential as a prognostic biomarker and therapeutic target. This research lays the groundwork for understanding the multifaceted roles and regulatory mechanisms of CAFs in HNSCC, thereby offering valuable perspectives for the development of targeted therapeutic strategies aimed at improving patient outcomes.

## 1. Introduction

Head and neck squamous cell carcinoma (HNSCC), a complex and heterogeneous group of malignancies, originates from various anatomical locations such as the oral cavity, oropharynx, and larynx [1]. The cancer ranks as one of the foremost causes of cancer-associated fatalities on a global scale, with startling statistics indicating nearly 650,000 new instances and around 330,000 deaths each year [2, 3]. Historically, the cornerstone of treatment modalities for HNSCC has been a triad consisting of surgical intervention, radiation therapy, and chemotherapy [4, 5]. In the contemporary landscape, immunotherapy has made significant inroads as a promising treatment paradigm, specifically targeting immune checkpoints like PD-1 and PD-L1 to reinvigorate the host's immune response against the cancer cells [4, 6, 7]. However, this optimism is tempered by the realization that only a fraction of patients displays a positive response to such immunotherapeutic interventions [4]. This limited efficacy has provoked critical questions and intensified the search for predictive biomarkers. Moreover, issues like the development of resistance to immune checkpoint inhibitors, the prohibitive costs associated with these cutting-edge treatments, and an array of adverse side effects, including autoimmune reactions, have further circumscribed their widespread application. Consequently, there is an unmet and urgent necessity to discover new prognostic markers and innovative therapeutic targets, all aimed at enhancing the clinical outcomes for individuals afflicted with this devastating disease.

The tumor microenvironment (TME) is a complex and dynamic ecosystem that consists of tumor cells, blood vessels, immune cells, and stromal cells, among which cancer-associated fibroblasts (CAFs) stand out for their multifaceted contributions to the progression and metastasis of HNSCC [8, 9]. CAFs are activated fibroblasts that have undergone phenotypic changes influenced by factors such as transforming growth factor-beta (TGF-*β*), platelet-derived growth factor (PDGF), and fibroblast growth factor (FGF) secreted by cancer cells and other stromal cells [10, 11]. One of the major roles of CAFs lies in the remodeling of the extracellular matrix (ECM), a lattice of proteins and polysaccharides that provides structural and biochemical support to surrounding cells. CAFs secrete various matrix metalloproteinases (MMPs), such as MMP2 and MMP9, that break down structural proteins like collagen and fibronectin, thereby making the ECM more amenable for tumor cell invasion [12]. Additionally, they produce an array of cytokines and chemokines, including IL-6, IL-8, and CXCL12, which can modulate immune responses and promote tumorigenesis. CAFs are prolific producers of angiogenic factors like vascular endothelial growth factor, thereby fostering the sprouting of new blood vessels from existing vasculature, a process crucial for tumor growth and metastasis [13, 14]. Clinical evidence suggests that a high density of CAFs within the tumor stroma is often correlated with poor prognosis and decreased survival rates in HNSCC patients [15]. Moreover, CAFs have been found to be involved in the epithelial–mesenchymal transition (EMT) [16], a process that augments the invasive and metastatic abilities of cancer cells.

Fibroblast activation protein (FAP) is a surface glycoprotein expressed by CAFs and has been implicated in multiple cancer types [17]. Functionally, FAP plays a vital role in ECM remodeling by cleaving-specific substrates like collagen and fibronectin. Overexpression of FAP has been reported to induce tumor cell proliferation, migration, and invasion [18]. It has also been implicated in angiogenesis and immunosuppression, further amplifying its potential as a therapeutic target [19]. However, the mechanistic underpinnings of FAP in HNSCC remain underexplored.

Given the pressing need for novel therapeutic strategies in HNSCC and the intriguing roles of CAFs and FAP in tumor biology, this study aims to elucidate the functional implications of FAP within the context of HNSCC. Utilizing a large-scale data analysis of The Cancer Genome Atlas (TCGA) HNSCC dataset, we performed differential gene expression analysis to identify CAF-associated genes. We then proceeded to validate the identified genes using quantitative polymerase chain reaction (qPCR) and western blot techniques. Furthermore, functional assays were carried out to examine the role of FAP in cellular proliferation, migration, and invasion. Gene Ontology (GO) and Kyoto Encyclopedia of Genes and Genomes (KEGG) enrichment analyses were conducted to identify the biological processes and pathways associated with FAP. In summary, this study endeavors to provide a comprehensive understanding of FAP's role in HNSCC, potentially offering new avenues for targeted therapies. By employing an integrated approach of bioinformatics, molecular biology, and functional assays, we aim to unravel the complex interactions between FAP, CAFs, and their downstream effectors in the pathogenesis and progression of HNSCC.

## 2. Materials and Methods

### 2.1. Data Processing

We acquired RNA-seq data and associated clinical information for 564 HNSCC cases from the TCGA database. Cases without clinical survival data were excluded. Data preprocessing and normalization were performed using the R packages “edgeR” and “DESeq2.” Differentially expressed genes (DEGs) were identified based on a *P*-value < 0.01 and a log2 fold change (FC) greater than 1 or less than −1. Kaplan–Meier survival analysis was utilized to assess the prognostic significance of CAFs-related mRNA levels in HNSCC.

### 2.2. Cell Culture and Experimental Design

HNSCC cell lines, procured from the China Center for Type Culture Collection, were cultured in DMEM medium (Gibco) and maintained at 37°C in a 5% CO_2_ atmosphere. For our experimental design, we meticulously categorized the study into two pivotal groups: the knockdown group and the overexpression group. In the knockdown group, targeted silencing of the FAP gene was achieved through transfection with specific FAP short hairpin RNA. The sequences were shRNA-1: ATCCCGTTGTTCGGATATTTA; shRNA-2: CCCTCAGACAGTTTGCTTATT. Conversely, the overexpression group involved the utilization of FAP gene (NM_004460) overexpression vectors (pCMV-FAP) for genetic enhancement. Both interventions were conducted using Lipofectamine™ 2000 (ThermoFisher) according to the manufacturer's guidelines.

### 2.3. Cell Proliferation Assay

Cell proliferation was assessed using the Cell Counting Kit-8 (CCK-8) reagent (DOJINDO). Cells were seeded in 96-well plates and cultured under standardized conditions. Absorbance measurements were conducted at 24, 48, 72, and 96-hr intervals postseeding.

### 2.4. Cell Migration Assay

The cell migration assay employed transwell chambers with 8 *μ*m pore-size inserts (Corning). Migrated cells on the lower filter surface were fixed with ice-cold methanol and stained with crystal violet for quantification.

### 2.5. Reverse Transcription qPCR (RT-qPCR) Analysis

Gene expression changes were evaluated using RT-qPCR. Total RNA was isolated using TRIzol reagent (Beyotime), followed by cDNA synthesis and quantitative PCR analysis to determine the expression levels of FAP and the reference gene GAPDH according to the manufacturer's guidelines (QIAGEN). The primer sequences are listed as follows:

  FAP (forward): 5′-CCAAAGACCCAGGAGCATATAG-3′;
  FAP (reverse): 5′-GTTTGTAGCCATCCTTGTCAC-3′.
  GAPDH (forward):5′-CCTGCACCACCAACTGCTTA-3′;
  GAPDH (reverse):5′-TCTTCTGGGTGGCAGTGATG-3′.

### 2.6. RNA-Sequencing Analysis

Total RNA was extracted from both the knockdown and control groups using Trizol reagent (Invitrogen, Carlsbad, CA, USA) following cell lysis. The integrity and concentration of the extracted RNA were assessed using the Agilent 2100 Bioanalyzer (Agilent Technologies, Santa Clara, CA, USA). Subsequently, ribosomal RNA was depleted from the total RNA samples using the Ribo-Zero rRNA Removal Kit (Illumina, San Diego, CA, USA). Complementary DNA (cDNA) was synthesized from the rRNA-depleted RNA using the SuperScript IV First-Strand Synthesis System (Invitrogen, Carlsbad, CA, USA). Following cDNA synthesis, library construction was performed using the NEBNext Ultra II DNA Library Prep Kit for Illumina (New England Biolabs, Ipswich, MA, USA), according to the manufacturer's instructions. The libraries were then quantified using the Qubit 2.0 Fluorometer (Life Technologies, Carlsbad, CA, USA) and validated for quality by the Agilent 2100 Bioanalyzer. The validated libraries were sequenced on an Illumina NovaSeq 6000 platform to generate paired-end reads of 150 bp in length. The RNA-seq dataset was uploaded to the GSA Database.

### 2.7. GO and KEGG Pathway Analysis

To elucidate the functional roles of the DEGs discovered via RNA-seq, we conducted GO and KEGG pathway analyses. The “clusterProfiler” R package was utilized for GO analysis to identify enriched biological processes, molecular functions, and cellular components, setting an adjusted *P*-value threshold of <0.05. Similarly, KEGG pathway analysis was conducted using the same package to identify significantly enriched pathways.

### 2.8. Patients Samples

HNSCC specimens and adjacent normal tissue samples from the Department of Oral and Maxillofacial Surgery at Shenzhen People's Hospital. We have obtained the ethical approval from Shenzhen People's Hospital's ethics committee. All patients provided informed consent and had not undergone preoperative radiotherapy or chemotherapy.

### 2.9. Western Blot Analysis

Protein extraction from cells in both the knockdown and control groups was performed using RIPA buffer (Beyotime), supplemented with protease and phosphatase inhibitors. The extracted proteins underwent SDS–PAGE for separation and were subsequently transferred to polyacrylamide gel membranes. After membrane transfer, the samples were blocked and incubated with anti-FAP (SAB, 36465), anti-LAMA3 (Abcam, ab242197), anti-SNAIL (Affinity Biosciences, AF6756), anti-SLUG (Cell Signaling Technology, 9585S) and anti-GAPDH antibodies (Proteintech, 60004). Chemiluminescence was utilized for the detection of protein expression, employing secondary antibodies (Proteintech, SA00001) for this purpose.

### 2.10. Apoptosis Analysis

Apoptosis assays were conducted using both flow cytometry and caspase-3 activity measurement. For the flow cytometry analysis, cells were stained with Annexin V-FITC and propidium iodide (PI) using the Annexin V Apoptosis Detection Kit I (BD Biosciences, San Jose, CA, USA) according to the manufacturer's instructions. Briefly, cells were collected, washed twice with cold PBS, and resuspended in 1x binding buffer. Then, 5 *µ*L of Annexin V-FITC and 5 *µ*L of PI were added to 100 *µ*L of the cell suspension, and the mixture was incubated for 15 min at room temperature in the dark. After the addition of 400 *µ*L of 1x binding buffer, the cells were analyzed by flow cytometry within 1 hr. Caspase-3 activity was measured using the Caspase-3/CPP32 Colorimetric Assay Kit (K106-100, BioVision, Milpitas, CA, USA), following the protocol provided by the manufacturer. Cells were lysed, and the lysates were incubated with the provided reaction buffer and caspase-3 substrate (DEVD-pNA) at 37°C for 2 hr. Absorbance was measured at 405 nm using a microplate reader to determine the enzymatic activity of caspase-3.

### 2.11. EMT Score Analysis

To evaluate the EMT status across various cancer cell lines, we computed EMT scores using the AddModuleScore function within the Seurat package in R. This method involves the aggregation of expression levels of key EMT markers to generate a score reflecting the mesenchymal phenotype. We further assessed the correlation between EMT scores and the expression profiles of other genes to identify potential biomarkers associated with EMT transitions. Pearson correlation coefficients were calculated to determine the statistical significance of associations, using a threshold of *P*  < 0.05 for significance. All analyses were conducted using R (version 4.0.2), ensuring rigorous and reproducible statistical evaluation.

### 2.12. Statistical Analysis

For pairwise comparisons, Student's *t*-test was utilized, while ANOVA was used for comparisons involving three or more groups. Statistical significance was set at *P* < 0.05. Data visualization and analysis were conducted using GraphPad Prism 8.0.

## 3. Results

### 3.1. Investigating the Functional Role of CAFs in HNSCC

**Figure 1:**
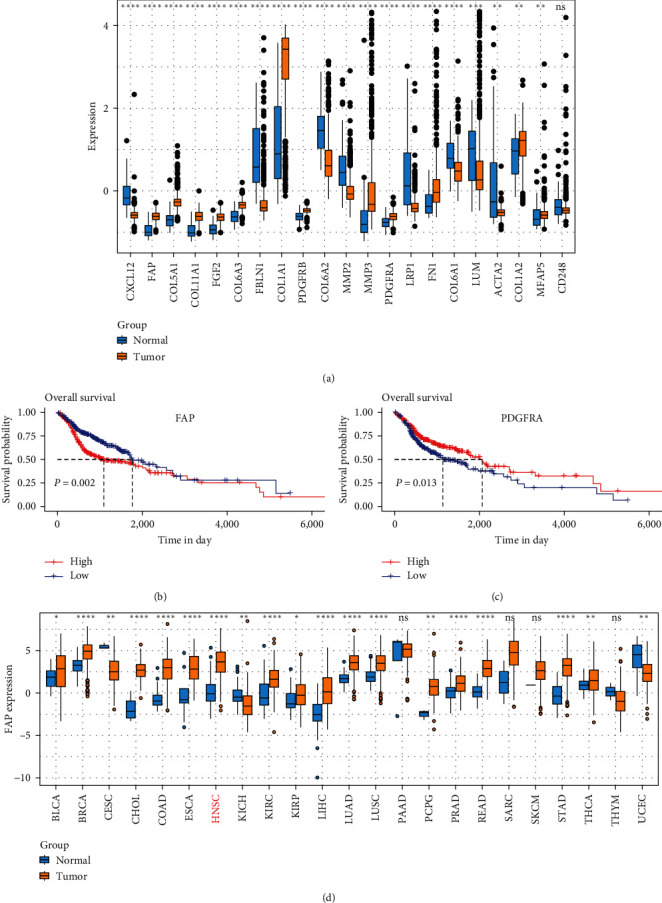
Comprehensive investigation of functional implications of CAFs in HNSCC. (a) Differential expression analyses of CAF-correlated genes, contrasting neoplastic with adjacent nontumoral tissues, derived from the TCGA HNSCC dataset. (b) Kaplan–Meier survival plots illustrate a statistically significant inverse correlation between elevated FAP expression and overall survival in HNSCC patients (*P*=0.002). (c) Kaplan–Meier survival plots disclose a significant inverse relationship between augmented PDGFRA expression and patient survival duration (*P*=0.013). (d) Pan-cancer analysis of FAP expression.  ^*∗*^*p* < 0.05,  ^*∗∗*^*p* < 0.01,  ^*∗∗∗*^*p* < 0.001, and  ^*∗∗∗∗*^*p* < 0.0001.

To interrogate the functional implications of gene signatures pertinent to CAFs in HNSCC [20] (Table 1), we performed a differential gene expression analysis contrasting tumoral and adjacent nontumoral tissues utilizing the TCGA HNSCC dataset. An abundance of genes, including FAP, COL1A1, COL5A1, and COL5A2, manifested a pronounced upregulation in neoplastic versus normal tissues (Figure 1(a)). In contrast, genes such as COL6A2, MMP2, and LRP1 were disproportionately expressed in the normal tissue (Figure 1(a)). To further clarify the clinical implications of CAF-correlated genes in HNSCC, we conducted survival analyses to investigate the association between individual gene expression patterns and patient outcomes. Our analysis revealed a statistically significant reduction in overall survival among patients with elevated FAP levels (*P*=0.002) (Figure 1(b)). Likewise, augmented expression of PDGFRA was significantly associated with reduced survival durations (*P*=0.013) (Figure 1(c)). Meanwhile, the prognostic value of other genes within this CAF-correlated gene set remained nonsignificant (Figure S1). Considering the pivotal role of FAP in cancer, we analyze its expression across 23 cancers using the TCGA database, revealing significant upregulation in 16 cancer types (specially in HNSCC), downregulation in three cancer types, with no significant changes observed in the other four cancers (Figure 1(d)). Besides, we analyzed the correlation between FAP expression and the expression profiles of typical immune checkpoint molecules (PD-1, CTLA4, LAG3, BTLA, CD274, HAVCR2, TIGIT, and CD276) simultaneously. We found that in HNSCC, FAP expression is significantly correlated with CTLA4, HAVCR2, and CD276 (Figure S2), suggesting that FAP may play a key role in regulating immune evasion and tumor immune suppression, but further exploration is needed to elucidate the specific mechanisms. Collectively, these data strongly suggest that FAP is a crucial molecular player in HNSCC pathogenesis and may serve as a robust prognostic biomarker.

### 3.2. Correlation between FAP Gene Expression and Clinical Characteristics in HNSCC

**Figure 2:**
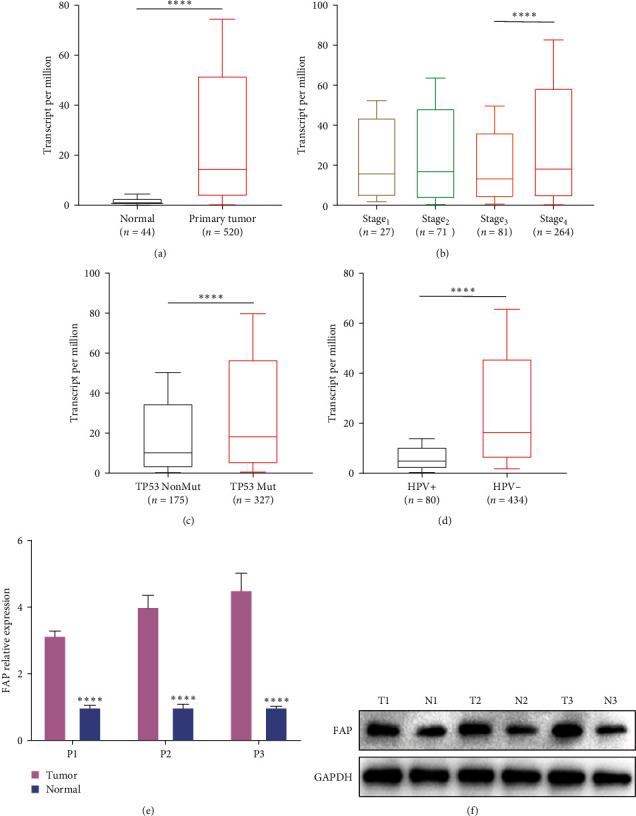
Expression of FAP in HNSCC and its correlation with clinical characteristics. (a) Comparative analysis of FAP expression between neoplastic and normal tissues within the TCGA HNSCC dataset. (b) Differential expression of FAP across disparate tumor stages in HNSCC patients. (c) Comparative FAP expression between TP53-mutated and nonmutated HNSCC patients. (d) Discrepancy in FAP expression contingent upon HPV status (HPV-positive versus HPV-negative) in HNSCC patients. (e) Quantitative PCR (qPCR) elucidation of differential FAP expression between tumoral and peritumoral tissues. (f) Validation of divergent FAP protein expression between neoplastic and adjacent non-neoplastic tissues in a cohort of three HNSCC patients, as ascertained by western blot analysis.  ^*∗∗∗∗*^*p* < 0.0001.

Within the TCGA HNSCC dataset, a comprehensive evaluation of FAP gene differential expression between malignant and normal tissues revealed a substantial upregulation in the oncogenic samples (Figure 2(a)). Stratified analysis across progressive disease stages identified a significant expression disparity between stages 3 and 4 (Figure 2(b)). Moreover, patients with TP53 mutations displayed a discernible elevation in FAP expression (Figure 2(c)). In a stark contrast, human papillomavirus (HPV)-positive HNSCC demonstrated a notable downregulation of FAP when compared to their HPV-negative counterparts (Figure 2(d)). Given the prevailing literature that supports a generally more benign phenotype for HPV-positive HNSCC [1, 21], these results fortify the hypothesis that FAP expression is intricately associated with the tumor's malignancy grade. To validate these findings, FAP expression was assayed in both tumoral and adjacent non-tumoral tissues in a representative cohort of three HNSCC patients. Employing qPCR, the data revealed that FAP expression in malignant tissues was notably amplified relative to adjacent non-tumoral tissues (Figure 2(e)). This observation was further corroborated through western blot analysis, confirming FAP overexpression within the oncogenic tissues across the triad of patients (Figure 2(f)).

### 3.3. FAP Mediates Cellular Proliferation and Apoptosis in HNSCC

**Figure 3:**
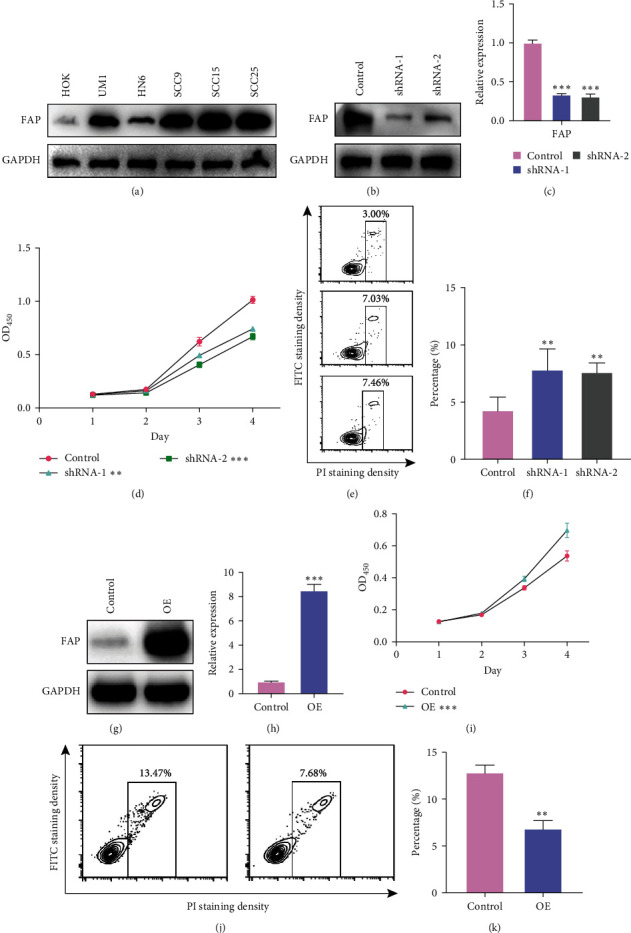
Regulatory role of FAP in cellular proliferation and apoptosis in HNSCC. (a) Western blot analysis across diverse HNSCC cell lines unveils elevated FAP expression in the SCC15 cell line. (b) Following FAP knockout in SCC15 cells, WB data clearly reveal a notable downregulation of FAP expression. (c) qPCR assays provide further evidence of the efficiency of FAP silencing in SCC15 cells. (d) A significant decline in SCC15 cell proliferation was observed post-FAP knockout, as confirmed by CCK8 assays. (e and f) Apoptotic status of SCC15 cells post-FAP knockout: (e) depicts a representative micrograph of apoptotic cells, and (f) offers a quantitative assessment of apoptosis. (g) WB data confirm a marked increase in FAP expression following its overexpression in HN6 cells. (h) qPCR assays validate the efficiency of FAP overexpression in HN6 cells. (i) FAP overexpression led to a noticeable enhancement in the proliferation of HN6 cells, as evidenced by CCK8 assays. (j and k) Apoptotic status of HN6 cells post-FAP overexpression: (j) shows a typical image of apoptotic cells, and (k) presents a quantitative analysis of apoptosis.  ^*∗∗*^*p* < 0.01 and  ^*∗∗∗*^*p* < 0.001.

To probe the functional roles of FAP in HNSCC, we first evaluated FAP expression across diverse HNSCC cell lines using western blot analysis (Figure 3(a)). Our findings unequivocally demonstrated elevated FAP expression in the SCC15 cell line (Figure 3(a)), thereby designating it as the focus for subsequent investigations. Genetic silencing of FAP in SCC15 cells led to a measurable reduction in its expression, as corroborated by Western Blot analysis (Figure 3(b)). This reduction was further confirmed via qPCR, which revealed a significant decline in FAP levels post-silencing (Figure 3(c)). Additionally, the CCK-8 assay indicated a significant decrease in the proliferation rate of FAP-deficient HNSCC cells compared to the control cells (Figure 3(d)). Simultaneously, we observed a marked increase in the percentage of apoptotic cells post-FAP silencing (Figures 3(e) and 3(f)). To reinforce these observations, we performed FAP overexpression assays in the HN6 cell line. Western blot analysis post-overexpression evidenced a notable upregulation in FAP levels (Figure 3(g)), a finding further substantiated by qPCR (Figure 3(h)). The CCK-8 assay confirmed that cells overexpressing FAP exhibited enhanced proliferative capabilities (Figure 3(i)), while the percentage of apoptotic cells significantly decreased upon FAP overexpression (Figures 3(j) and 3(k)). Collectively, these results underscore FAP's regulatory role in both the cellular proliferation and apoptotic pathways of HNSCC.

### 3.4. Investigating the Role of FAP in the Progression of HNSCC and Its Associated Pathways

**Figure 4:**
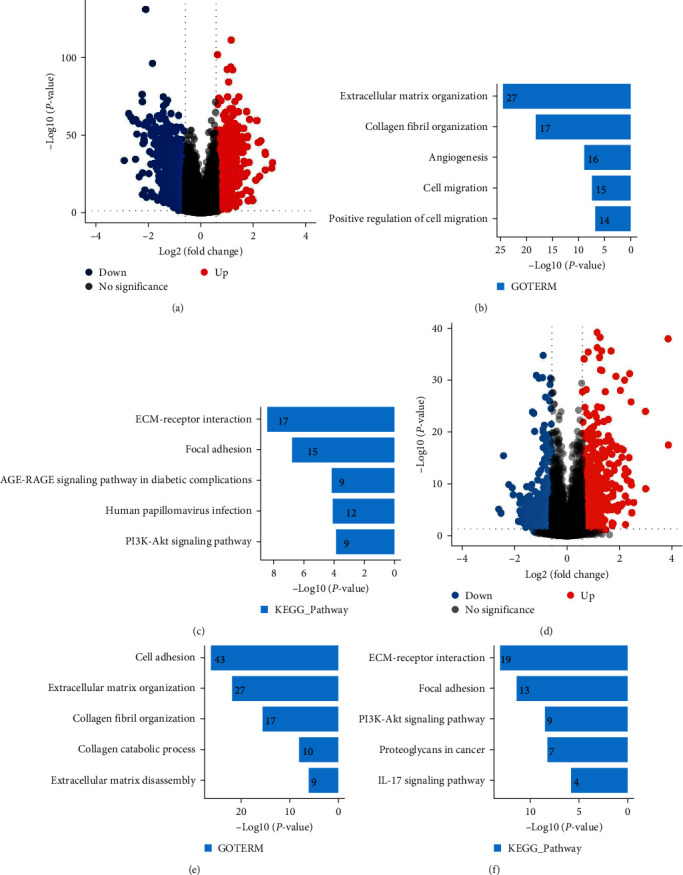
Exploring the impact of FAP on HNSCC progression and associated pathways. (a) A differential gene expression volcano plot, generated from the TCGA HNSCC dataset and dividing patients into cohorts with high and low FAP expression, sharply identifies the distinct characteristic genes between the two groups. (b) GO pathway enrichment analysis highlights the relationship between increased FAP expression and key biological processes. (c) KEGG pathway enrichment outcomes clarify the strong correlation between elevated FAP levels and signaling pathways. (d) RNA-Seq profile graphs for both SCC15 control and knockout cells. (e) Results demonstrate a marked reduction in pathways associated with cellular adhesion, extracellular matrix structuring, collagen fibril formation, collagen breakdown, and matrix degradation following FAP removal. (f) KEGG analysis reveals considerable downregulation in pathways concerning ECM-receptor binding, focal adherence, PI3K-Akt signaling, cancer proteoglycans, and IL-17 signaling post-FAP ablation.

To explore more exhaustively the mechanistic underpinnings of how FAP contributes to the development and progression of HNSCC, we utilized patient data from the TCGA HNSCC dataset. Patients were classified into two distinct cohorts based on high or low FAP expression levels. We performed differential gene expression analysis, encapsulated in a volcano plot (Figure 4(a)), which distinctly outlines the distinguishing genes between these two cohorts. GO enrichment analysis identified that elevated FAP expression strongly correlates with vital biological functions such as ECM organization, collagen fibril formation, angiogenesis, and cellular motility (Figure 4(b)). KEGG enrichment analysis further validated the significant association between high FAP expression and pathways like ECM-receptor interactions, focal adhesions, AGE-RAGE signaling in diabetes-related complications, HPV infection, and the PI3K-Akt signaling cascade (Figure 4(c)). To corroborate these findings, we conducted RNA-Seq experiments on both SCC15 control and SCC15 knockout cells (Figure 4(d)). The data elucidated that FAP ablation leads to notable decreases in pathways linked to cell adhesion, ECM integrity, collagen dynamics, and ECM degradation (Figure 4(e)). Concurrent KEGG analysis supported the significant downregulation of pathways associated with ECM-receptor interactions, focal adhesions, PI3K-Akt signaling, proteoglycans in cancer, and IL-17 signaling post-FAP ablation (Figure 4(f)). Overall, these comprehensive results substantiate the notion that FAP likely augments the malignant behavior of HNSCC by influencing the ECM architecture.

### 3.5. Implications of FAP on the Expression of Genes Related to ECM Formation and Cellular Invasiveness

**Figure 5:**
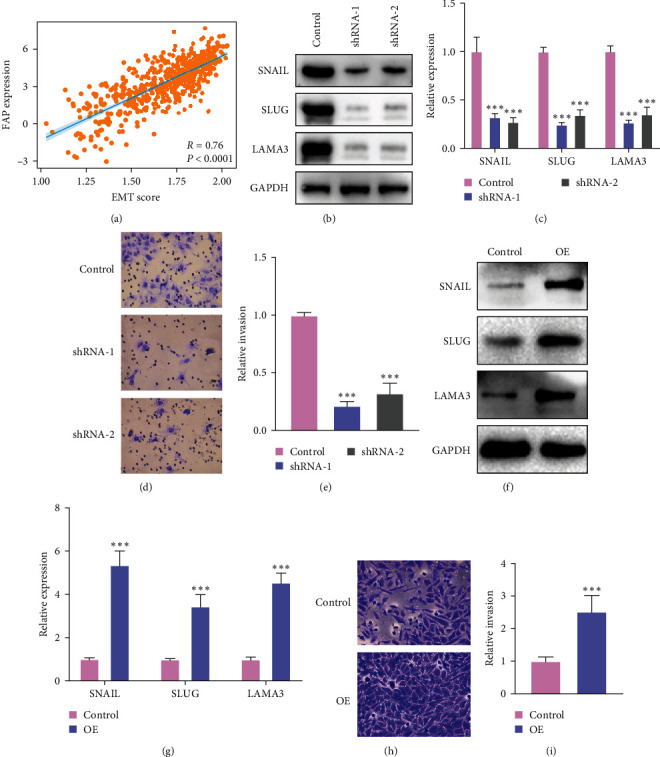
Deciphering the role of FAP in extracellular matrix regulation and EMT process through key mediators. (a) The correlation analysis between FAP expression and EMT score. (b) Western blot results showed a noteworthy downregulation of SNAIL1, SLUG, and LAMA3 after the knockout of FAP in SCC15 cells. (c) The reduction was further verified through qPCR assessments. (d and e) Transwell invasion assays confirmed a pronounced decrease in the cells' invasive capacity following FAP removal. (f) Conversely, the overexpression of FAP led to a marked increase in the levels of SNAIL1, SLUG, and LAMA3, as demonstrated by western blot assays. (g) The upregulation was further substantiated using qPCR tests. (h and i) Complementary transwell invasion assays on the control, and FAP-overexpressed groups revealed a significant escalation in cellular invasiveness after FAP augmentation.  ^*∗∗∗*^*p* < 0.001.

Given the close association between the ECM and the EMT signaling pathway [22, 23], our study delves into the pivotal role of FAP in modulating ECM dynamics and EMT progression in HNSCC. We found FAP expression correlates significantly with EMT scores, indicating its substantial involvement in EMT processes for HNSCC progression (Figure 5(a)). In light of existing scholarly evidence implicating proteins SNAIL1, SLUG, and LAMA3 in the modulation of the ECM and EMT process [24–26], we further investigated the role of FAP in its architecture and remodeling. To this end, we examined the expression levels of SNAIL1, SLUG, and LAMA3 in the SCC15 cell line subjected to FAP knockout. Western blot assays demonstrated a pronounced downregulation of these proteins following the elimination of FAP (Figure 5(b)), which was substantiated by qPCR analyses (Figure 5(c)). Complementary transwell invasion assays indicated a considerable attenuation of cellular invasiveness post-FAP ablation (Figure 5(d) and 5(e)). As a converse approach, we scrutinized gene expression in HN6 cells with FAP overexpression. This overexpression induced a discernible upregulation of SNAIL1, SLUG, and LAMA3 (Figure 5(f)), corroborated by subsequent qPCR analyses (Figure 5(g)). Parallel transwell invasion assays demonstrated a consequential elevation in cellular invasiveness in the overexpression cohort (Figures 5(h) and 5(i)). Collectively, these findings suggest that FAP plays a pivotal role in the pathogenesis and progression of HNSCC by regulating the structural and functional dynamics of the ECM and EMT process.

## 4. Discussion

CAFs constitute a heterogeneous mesenchymal cell population that significantly influences the TME, thereby aiding in tumorigenesis, metastasis, and resistance to therapy [10, 27–29]. A hallmark of CAFs is FAP [30, 31], a cell surface serine protease essential for remodeling the ECM, which in turn promotes tumor cell invasion and migration [32]. Beyond this, FAP regulates critical signaling pathways affecting both neoplastic and stromal cells, thereby enhancing angiogenesis, facilitating immune evasion, and promoting metabolic reprograming within the tumor milieu [33, 34]. Consequently, FAP serves as both a robust marker for CAFs identification and a promising therapeutic target to disrupt tumor-stroma interactions and counter tumor progression.

Our exhaustive analysis of the TCGA HNSCC dataset emphasizes FAP's critical role in the pathogenesis and clinical course of HNSCC. Additionally, our analysis revealed a significant correlation between FAP expression and the expression profiles of typical immune checkpoint molecules (CTLA4, HAVCR2, and CD276). However, further investigation is warranted to elucidate the specific mechanisms underlying this association. We identified a pronounced overexpression of FAP in cancerous tissues relative to normal tissues, consistent across various disease stages and markedly accentuated in TP53-mutant specimens. Elevated FAP levels were significantly correlated with poorer overall survival, solidifying its utility as a prognostic biomarker. In vitro studies using SCC15 cell lines corroborated FAP's involvement in cellular proliferation, apoptosis, and invasiveness, underscoring its influence on tumor aggressiveness.

The GO and KEGG pathway analyses provide robust evidence supporting FAP's central role in coordinating essential biological processes and molecular pathways, particularly those governing ECM-receptor interactions and focal adhesions. These analytical insights shed light on the downstream signaling pathways influenced by FAP, suggesting its potential involvement in driving the EMT process. By elucidating the intricate interplay between FAP and key signaling pathways regulating EMT, our findings transcend mere observations of FAP gene expression, highlighting its profound impact on the TME. This transformative effect ultimately fuels cancer progression, thereby underscoring FAP as a promising target for cancer therapeutic intervention. Moreover, the regulatory influence of FAP extends to key proteins such as SNAIL1, SLUG, and LAMA3. SNAIL1 and SLUG, both zinc-finger transcription factors, are instrumental in EMT, a process integral to tumor metastasis [35]. LAMA3, a constituent of laminin-332, is involved in cellular adhesion and migration [36]. These proteins synergize under FAP's regulatory scope to complexly modulate ECM dynamics, thereby supporting an aggressive tumor phenotype and facilitating cancer dissemination. Hence, FAP's interaction with these proteins not only amplifies its regulatory complexity but also accentuates its candidacy as a therapeutic target for disrupting these multifaceted tumor–stroma interactions.

Our study enhances current understanding by both identifying and mechanistically validating FAP's role in HNSCC. While insightful, the research has limitations. Further investigations are needed to explore FAP's relationship with other potential biomarkers and its interactions with various cellular components within the TME. Although FAP emerges as a compelling therapeutic target, subsequent animal studies and clinical trials are essential to translate these findings into clinically relevant interventions. Collectively, our data suggest FAP is not only a mechanistic cornerstone in HNSCC pathogenesis but also a potential focus for future targeted therapies to improve patient outcomes in HNSCC.

## Figures and Tables

**Table 1:** The 21 CAFS-related genes (related to Figure 1).

Gene	Gene signature	PMID
*ACTA2*	Cancer-associated fibroblasts	21984967
*CD248*	Cancer-associated fibroblasts	18187565
*COL11A1*	Cancer-associated fibroblasts	27021528
*COL1A1*	Cancer-associated fibroblasts	16572188
*COL1A2*	Cancer-associated fibroblasts	16572188
*COL5A1*	Cancer-associated fibroblasts	16572188
*COL6A1*	Cancer-associated fibroblasts	26338964
*COL6A2*	Cancer-associated fibroblasts	26338965
*COL6A3*	Cancer-associated fibroblasts	26338966
*CXCL12*	Cancer-associated fibroblasts	26414794
*FAP*	Cancer-associated fibroblasts	22323494
*FBLN1*	Cancer-associated fibroblasts	22482059
*FGF2*	Cancer-associated fibroblasts	25973543
*FN1*	Cancer-associated fibroblasts	26068592
*LRP1*	Cancer-associated fibroblasts	21325077
*LUM*	Cancer-associated fibroblasts	28542982
*MFAP5*	Cancer-associated fibroblasts	29681158
*MMP2*	Cancer-associated fibroblasts	23705892
*MMP3*	Cancer-associated fibroblasts	23705892
*PDGFRA*	Cancer-associated fibroblasts	27881889
*PDGFRB*	Cancer-associated fibroblasts	27881889

## Data Availability

The data that support the findings of this study are available on reasonable request from the corresponding author, Yuyan Zheng.

## Supplementary Materials

Supplementary Materials Figure S1: Kaplan–Meier survival plots elucidate the prognostic relevance of additional genes within the CAF-correlated gene set in HNSCC. Figure S2: the relationship between FAP and relevant immune checkpoints.
