# Supplementary material for: FAP Serves as a Prognostic Biomarker in Head and Neck Squamous Cell Carcinoma
Source: Anal Cell Pathol (Amst). 2024 May 24;2024:8810804. doi: 10.1155/2024/8810804 (PMC11142855; doi:10.1155/2024/8810804)
Supplement: Supplementary Materials — Figure S1: Kaplan–Meier survival plots elucidate the prognostic relevance of additional genes within the CAF-correlated gene set in HNSCC. Figure S2: the relationship between FAP and relevant immune checkpoints. [file 8810804.f1.docx]

**Figure S1**


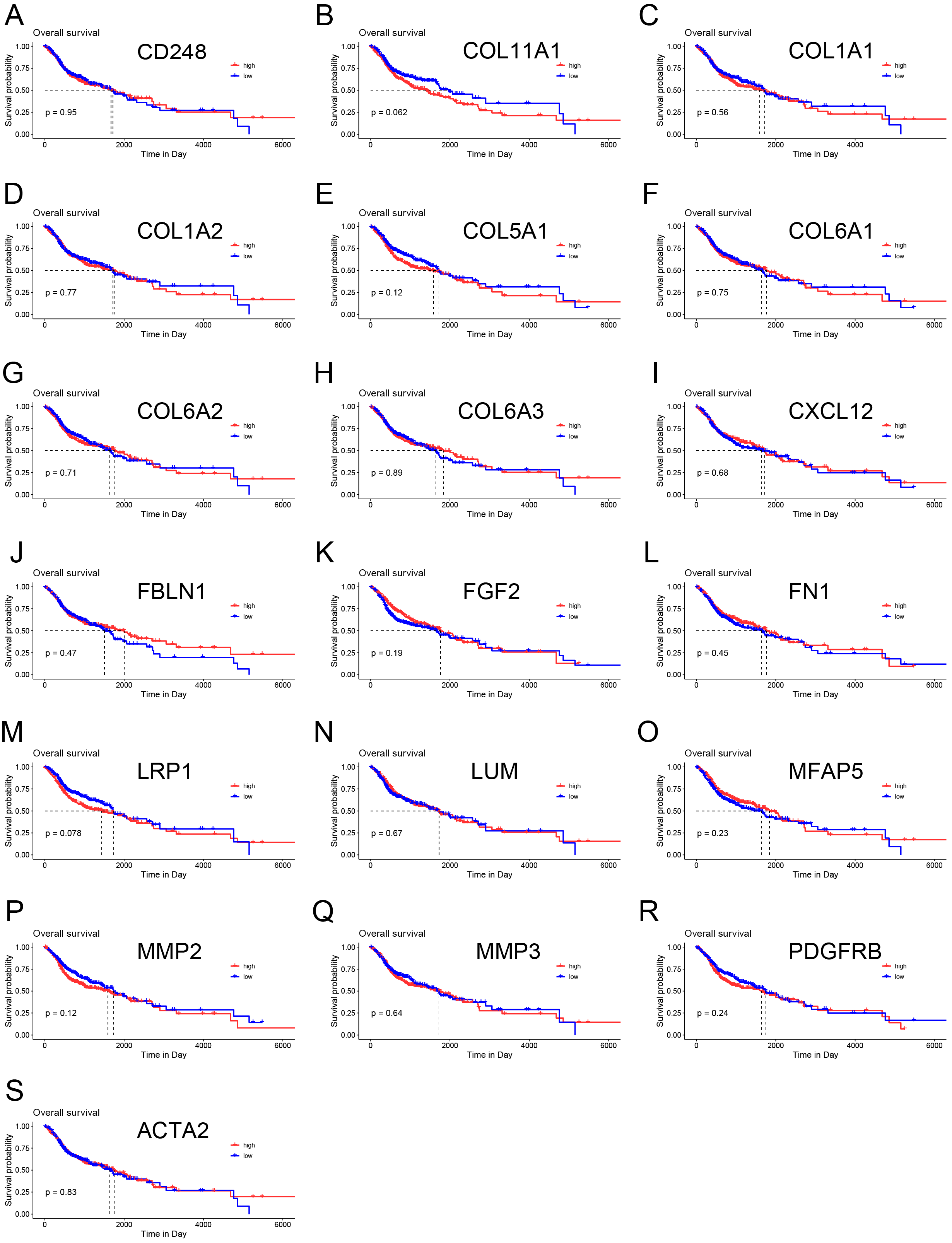


Supplementary Figure 1. Kaplan-Meier survival plots elucidate the prognostic relevance of additional genes within the CAF-correlated gene set in HNSCC. The analysis reveals the absence of a statistically significant association between the expression of these genes and patient survival outcomes.

**Figure S2**


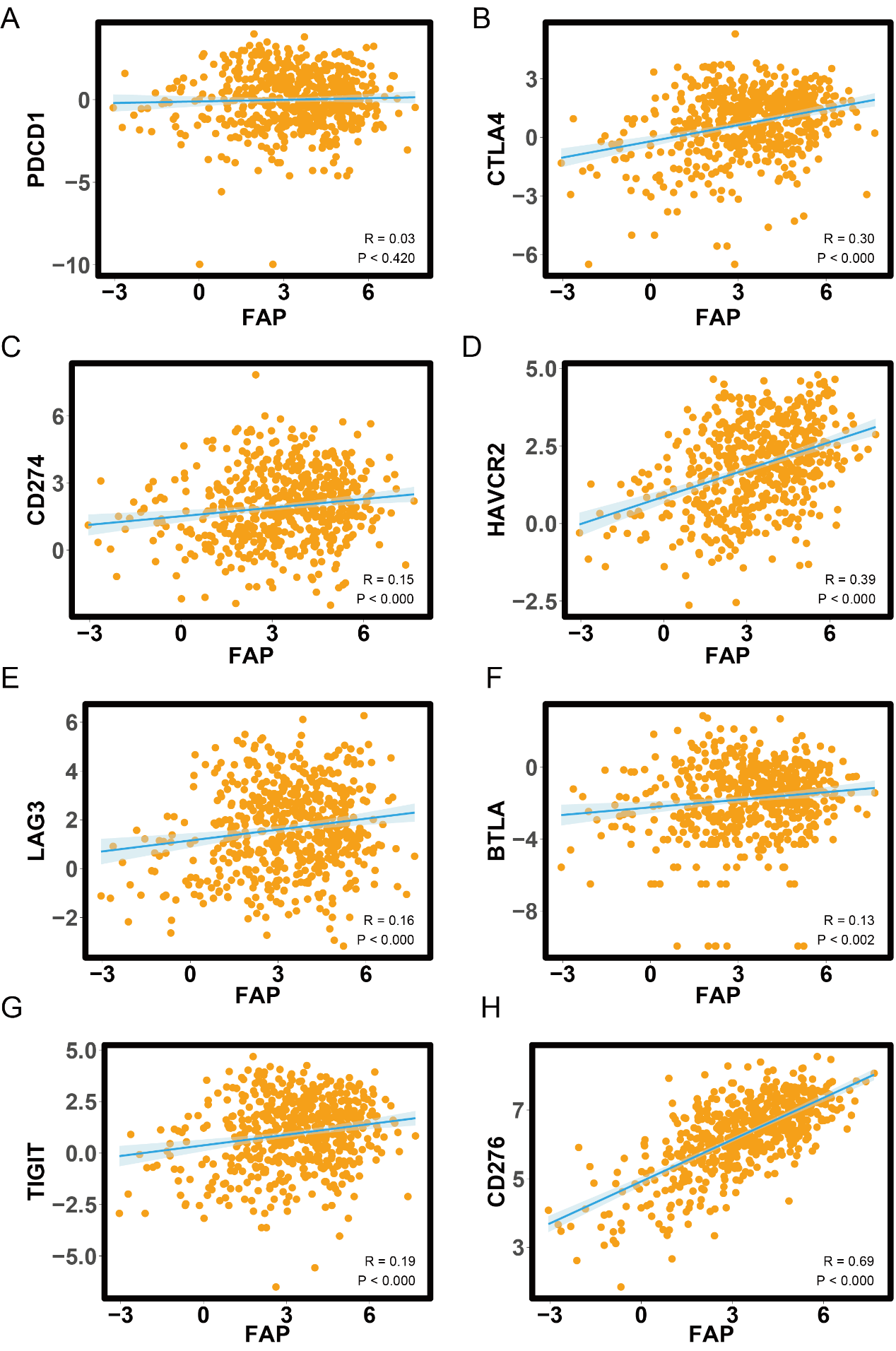


Supplementary Figure 2. The relationship between FAP and relevant immune checkpoints. The correlation analysis between FAP expression and typical immune checkpoints (PD-1, CTLA4, LAG3, BTLA, CD274, HAVCR2, TIGIT, CD276).
